# Network Pharmacology Study and Experimental Validation of Yiqi Huayu Decoction Inducing Ferroptosis in Gastric Cancer

**DOI:** 10.3389/fonc.2022.820059

**Published:** 2022-02-14

**Authors:** Siyuan Song, Fang Wen, Suping Gu, Peixin Gu, Wenjie Huang, Shuai Ruan, Xiaoxue Chen, Jiayu Zhou, Ye Li, Jiatong Liu, Peng Shu

**Affiliations:** ^1^ Department of Medical Oncology, Affiliated Hospital of Nanjing University of Chinese Medicine, Nanjing, China; ^2^ The First College for Clinical Medicine, Nanjing University of Chinese Medicine, Nanjing, China; ^3^ Department of Medical Oncology, Jiangsu Provincial Hospital of Chinese Medicine, Nanjing, China

**Keywords:** ferroptosis, GC, network pharmacology, molecular docking, bioinformatics, Yiqi Huayu decoction

## Abstract

**Objective:**

This study aimed to identify the mechanism of Yiqi Huayu Decoction (YQHY) induced ferroptosis in gastric cancer (GC) by using network pharmacology and experimental validation.

**Methods:**

The targets of YQHY, ferroptosis-related targets, and targets related to GC were derived from databases. Following the protein–protein interaction (PPI) network, the hub targets for YQHY induced ferroptosis in GC were identified. Furthermore, gene ontology (GO) and Kyoto Encyclopedia of Genes and Genomes (KEGG) enrichment were used to analyze the hub targets from a macro perspective. We verified the hub targets by molecular docking, GEPIA, HPA, and the cBioPortal database. Finally, we performed cell viability assays, quantitative real-time polymerase chain reaction (qRT-PCR), western blotting, lipid peroxidation, and GSH assays to explore the mechanism of YQHY induced ferroptosis in GC.

**Results:**

We identified the main active compounds and hub targets: Quercetin, DIBP, DBP, Mipax, Phaseol and TP53, ATM, SMAD4, PTGS2, and ACSL4. KEGG enrichment analyses indicated that the JAK2-STAT3 signaling pathway may be a significant pathway. Molecular docking results showed that the main active compounds had a good binding activity with the hub targets. The experimental results proved that YQHY could induce ferroptosis in AGS by increasing the MDA content and reducing the GSH content. qRT–PCR and Western blot results showed that YQHY can induce ferroptosis in GC by affecting the JAK2-STAT3 pathway and the expression of ACSL4.

**Conclusions:**

This study indicated that YQHY can induce ferroptosis in GC by affecting the JAK2–STAT3 pathway and the expression of ACSL4, and induction of ferroptosis may be one of the possible mechanisms of YQHY’s anti-recurrence and metastasis of GC.

## 1 Introduction

The incidence of GC in China accounts for nearly half of the world (1, 2). At present, surgery is considered the only radical cure method (3). Early GC has a high cure rate, whereas advanced GC is characterized by a high metastasis rate, a high mortality rate, a low curative resection rate, and a poor five-year survival rate (4). The occurrence and development of cancer are closely related to cell death. One of the important characteristics of cancer cells is avoiding death. Thus, it is necessary to explore a new strategy to treat GC by inducing tumor cell death.

Ferroptosis is a new cell death mode discovered in recent years, which is different from autophagy, necrosis, and scorch death. It is the result of ferroptosis-dependent accumulation of lipid peroxides to lethal levels (5). The ferroptosis signature is mainly characterized by the accumulation of iron and lipid reactive oxygen species and inhibition of the cystine/glutamate antiporter (system XC), leading to reduced cystine uptake and reduced glutathione (GSH) synthesis (6). Studies have shown that ferroptosis plays an important role in the development of various cancers (7). There is a close relationship between ferroptosis and GC, as ferroptosis is significantly suppressed in GC, resulting in reduced tumor growth and sensitivity to cisplatin versus paclitaxel (8). The expression of ultralong-chain fatty acid protein 5 and fatty acid desaturase 1 in mesenchymal GC cells is upregulated, leading to ferroptosis sensitization (9). Erastin, a typical ferroptosis inducer, can induce ferroptosis in GC cells. Silencing CDO1 *in vitro* and *in vivo* can restore intracellular glutathione levels and prevent ROS production, thus inhibiting ferroptosis in GC cells induced by erastin (10, 11). Therefore, inducing GC cells to undergo ferroptosis could be a potential direction for GC treatment (12).

**Figure 1:**
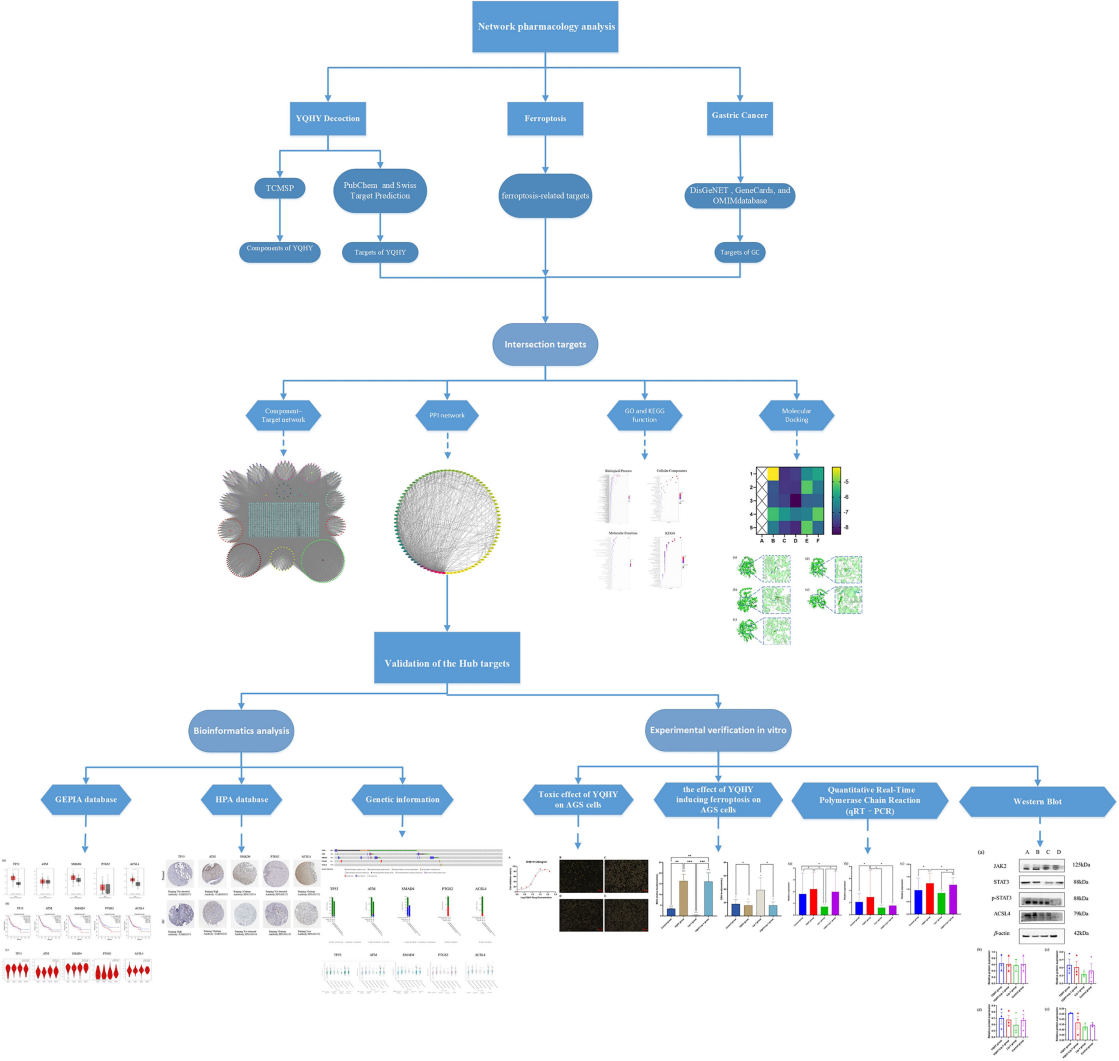
The protocol of our study procedures. (*P < 0.05, ** P < 0.01, *** P < 0.001).

The pathogenesis of postoperative patients with GC can be summarized as “Qi deficiency and blood stasis and toxin”, with deficiency of vital qi as the foundation and blood stasis and toxin as the standard. YQHY was pioneered by Professor Shen Lin Liu of a nationwide Chinese medicine and is based on Invigorating Qi and invigorating spleen and removing blood stasis as therapeutic points for GC, which is widely used in the clinical treatment of GC. Our previous studies have proven that YQHY can reduce the risk of recurrence and metastasis of patients with GC after stage II and III operations. Compared with chemotherapy alone, the risk of recurrence and metastasis is decreased by 32.8% (P = 0.0042). Especially for patients with stage III disease, the percentage of recurrence and metastasis risk is decreased to 34.7% (P = 0.0072) (13). Traditional Chinese medicine (TCM) has “multicomponent, multitarget, and multipathway” advantages for treating diseases (14). However, the mechanism of YQHY in reducing the risk of recurrence and metastasis of GC is still unclear. Therefore, in this paper, we combined the results of the network pharmacology study and verified them through experiments *in vitro*, which can provide new ideas for clarifying the pathogenesis of GC and for clinical intervention and treatment. The protocol of our study procedures is shown in **Figure 1**.

## 2 Methods

### 2.1 Network Pharmacology Analysis

#### 2.1.1 Screening the Main Active Compounds of YQHY

The active compounds of YQHY were derived from the TCMSP database (TCMSP, http://lsp.nwu.edu.cn/tcmsp.php). To better screen the active compounds of YQHY, OB ≥30% (15) and DL ≥0.05 (16) were used as screening conditions in this article. After that, a total of 360 active compounds were finally obtained.

#### 2.1.2 Collection of Targets

Through the PubChem database (https://pubchem.ncbi.nlm.nih.gov/), the screened active compounds were transformed into the SMILES structural formula, and the SMILES structural formula was imported into the Swiss Target Prediction website, setting *Homo sapiens*, to predict all potential targets of the active compounds in YQHY. The relevant targets in GC were obtained by using the DisGeNET, GeneCards, and OMIM databases. We screened targets related to ferroptosis from the FerrDB (http://www.zhounan.org/ferrdb/) database (17). All potential targets and corresponding active compounds of YQHY were input into Cytoscape to construct the “YQHY active compound-target” network.

#### 2.1.3 Screening of Hub Targets for YQHY Induced Ferroptosis in GC

Taking the intersection of the whole decoction targets, GC targets, and ferroptosis targets, we obtained the targets of YQHY induced ferroptosis in GC. The targets were imported into the STRING database to construct a PPI network and visualized using Cytoscape, of which the CytoNCA plug-in was used to calculate the parameters, and the hub targets for YQHY induced ferroptosis in GC were obtained according to BC, CC, and Degree.

#### 2.1.4 GO and KEGG Enrichment Analysis

The targets of YQHY induced ferroptosis in GC were imported into the DAVID database for GO and KEGG enrichment analysis. The final analysis results included

Biological Process (BP), Cellular Component (CC), and Molecular Function (MF). GO enrichment analysis was performed according to P <0.05 and FDR <0.05, and KEGG enrichment analysis was performed according to P <0.01 as the filtering criterion. Top20 were plotted into bubble charts using R language.

#### 2.1.5 Molecular Docking

Molecular docking is mainly used for structural docking of small molecules with target proteins and evaluating their binding affinities with defined binding sites (18). A negative docking binding energy suggests efficient autonomous binding of the small molecule to the target protein. It is generally believed that the lower the energy the conformation in which a ligand binds to a receptor is stabilized, the more likely the effect will be. In this study, the main active compounds and hub targets of YQHY induced ferroptosis in GC were molecularly docked. The TCMSP database was used to download the structure diagram of the main active compounds, which were saved in SDF format. Open Babel GUL software was used to convert the SDF format into the PDB format. We used the PDB database to download the 3D structure diagram of hub targets, and saved it in PDB format, then imported them into AutoDock software for molecular docking. Docking results were visualized using PyMol software.

### 2.2 Bioinformatics Analysis

The hub targets were input into the online tool GEPIA (http://gepia.cancer-pku.cn/index.html) (19) to verify their mRNA expression level, pathological stages, and overall survival (OS) in TCGA-STAD. The protein expression of hub targets was investigated in the HPA database (https://www.proteinatlas.org/) (20). The cBioPortal tool (http://www.cbioportal.org/) (21) was used to discover the genetic information and the correlation between mRNA expression of hub targets.

### 2.3 Experimental Validation *In Vitro*

#### 2.3.1 Preparation of YQHY Freeze-Dried Powder

The YQHY decoction consists of Huangqi, Dangshen, Chenpi, Banxia, Baizhu, Baishao, Danggui, Sanleng, Ezhu, Sheshecao, Shijianchuan, Fulin, Muxiang, Sharen, and Gancao. YQHY medicine was dried for 24 h, pulverized, and sieved with an 80 mesh sieve. The decoction was mixed, filtered, and concentrated to 250 ml. The concentrated solution was added to a special freeze-drying bottle, quickly frozen with liquid nitrogen, and dried at −50°C in FreezeDryers. The lyophilized powder was removed at 48 h and stored under cryogenic drying after crushing.

#### 2.3.2 Cell Viability Assay

Different concentrations of YQHY were added to the experimental group, and F12K culture medium was added to the zeroing group and control group. The reaction was carried out at 37°C and 5% carbon dioxide for 24 h. After CCK8 solution was added to each well to react for 1 h, the OD of each sample was measured by setting up the microplate reader at 490 nm. IC50 was calculated using GraphPad Prism software.

#### 2.3.3 Lipid Peroxidation and GSH Assays

The MDA and GSH contents in cell lysates were assessed using lipid peroxidation and GSH assay kits, respectively, according to the manufacturer’s (Abcam) instructions.

#### 2.3.4 Quantitative Real-Time Polymerase Chain Reaction (qRT-PCR)

According to the results of network pharmacology, we selected ACSL4, JAK2, and STAT3 to explore the mechanism by which YQHY induces ferroptosis in GC. Total RNA was extracted from cultured cells using TRIzol and phenol–chloroform phase separation according to the manufacturer’s instructions and then reverse transcribed using the RevertAid RT Reverse Transcription Kit (Invitrogen, K1691). The primer sequences used for qRT-PCR are listed in **Table 1**. qRT–PCR was performed using a StepOne Real-Time PCR system (Applied Biosystems).

**Table 1:** Real-Time polymerase chain reaction primers.

Gene	Sequence (5′-3′)
JAK2-F	CGAATGGTGTTTCTGATGTACC
JAK2-R	GTCTCCTACTTCTCTTCGTACG
STAT3-F	TTGTGTGTATGCGTCGGCTTCAG
STAT3-R	GCGGCTATACTGCTGGTCAATCTC
ACSL4-F	TCTCTTGCCTCAGCCTCCTTAGTAG
ACSL4-R β-ACTIN-F	CGAGACCAGCCTGACCAACATG CAGATGTGGATCAGCAAGCAGGA
β-ACTIN-R	CGCAACTAAGTCATAGTCCGCCTA

#### 2.3.5 Western Blot

The protein expression of JAK2, STAT3, p-STAT3, and ACSL4 was detected by Western blot. Total proteins were obtained using RIPA buffer, and protein concentrations were measured using a BCA protein assay kit. The protein samples were blotted onto PVDF membranes. Next, the membranes were incubated at 4°C overnight with primary antibodies against JAK2, STAT3, p-STAT3, ACSL4, and β-actin, followed by HRP-conjugated secondary antibodies. The grayscale values were quantified by Image Lab software.

#### 2.3.6 Statistical Analysis

The experimental results were statistically analyzed by GraphPad Prism software. Single-factor ANOVA was used to compare the differences in experimental data among groups. A P-value less than 0.05 was considered significant.

## 3 Results

### 3.1 Network Pharmacology Analysis

#### 3.1.1 Screening the Main Active Compounds of YQHY

Through the screening of the TCMSP database, a total of 360 active compounds were obtained, namely, 23 Huangqi, 27 Dangshen, 10 Chenpi, 20 Banxia,16 Baizhu, 17 Baishao, 22 Danggui, 10 Sanleng, 21 Ezhu, 7 Sheshecao, 4 Shijianchuan, 16 Fulin, 30 Muxiang, 54 Sharen, and 83 Gancao.

#### 3.1.2 Collection of Targets and Construction of the “YQHY compound-target” Network

**Figure 2:**
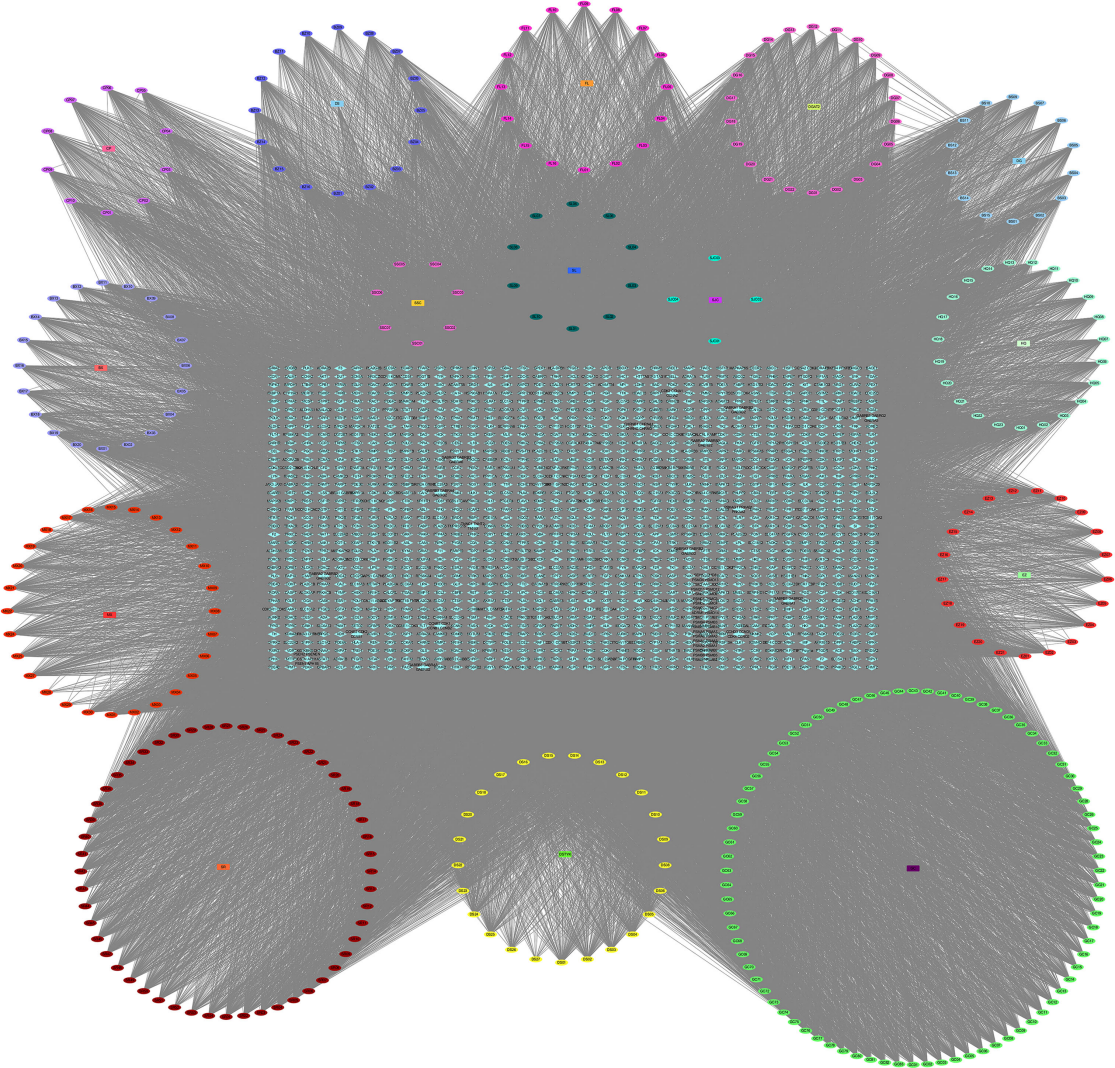
YQHY Compound-Target Network. The circle represents the YQHY active compound, the rectangle represents the herb, and the blue diamond represents the target.

**Figure 3:**
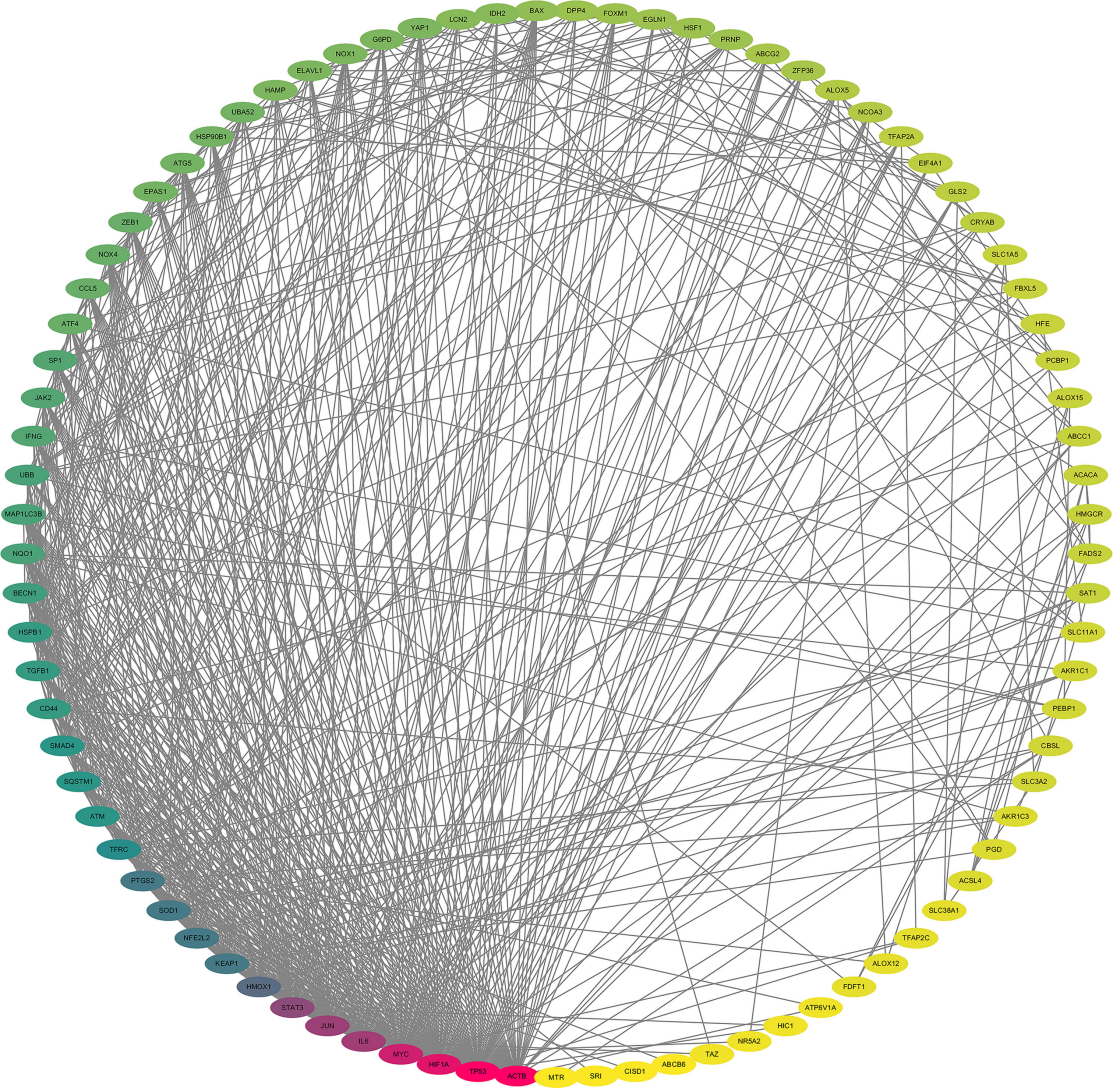
Protein–protein interaction Network. The darker the color, the greater the Degree value. The lighter the yellow color, the smaller the degree value.

After the prediction of the database and deletion of duplicate values, a total of 1,373 potential targets of active compounds, 15,842 targets of GC, and 205 targets of ferroptosis were obtained. A total of 87 common targets were obtained by combining the three target sets. At the same time, a “YQHY compound-target” network was constructed (**Figure 2**), in which quercetin, DIBP, DBP, Mipax, and Phaseol were considered the main active components for YQHY induced ferroptosis in GC. Then the PPI network was constructed according to the degree value (**Figure 3**).

#### 3.1.3 Screening of Hub Targets for YQHY Induced Ferroptosis in GC

According to the ranking of BC, CC, and degree calculated by the CytoNCA plug-in, TP53, ATM, SMAD4, PTGS2, and ACSL4 were identified as the hub targets of YQHY induced ferroptosis in GC. Meanwhile, a PPI network diagram of hub targets was constructed.

#### 3.1.4 GO and KEGG Enrichment Analysis

**Figure 4:**
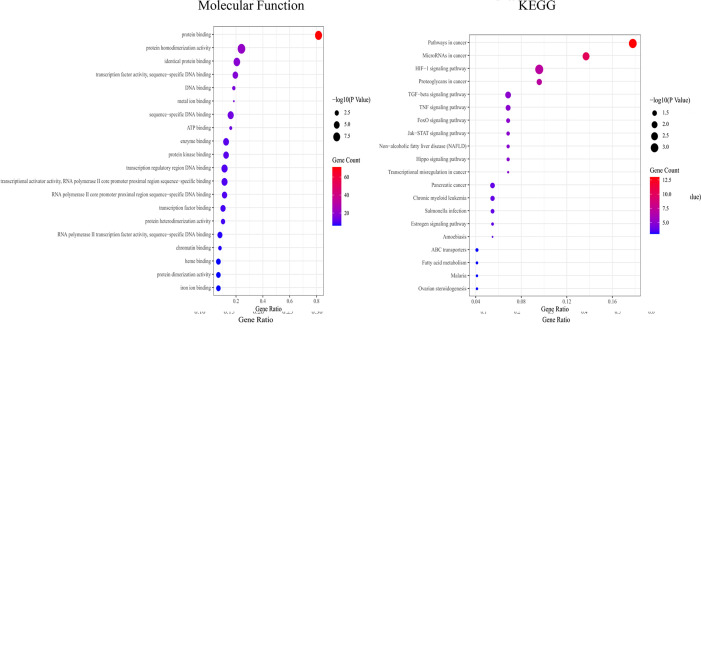
Bubble diagram for GO and KEGG enrichment analysis. The bubble size represents the number of enriched genes, and the bubble color difference represents the significant magnitude of target gene enrichment.

GO enrichment analysis showed that the biological functions mainly involved positive regulation of transcription from the RNA polymerase II promoter, negative regulation of apoptotic processes, and oxidation–reduction processes. Cell components mainly included the cytoplasm, cytosol, and nucleus. Molecular functions mainly included protein binding, identical protein binding, and DNA binding. KEGG functional enrichment analysis showed that the hub targets were mainly enriched in microRNAs in cancer and the JAK2-STAT3 signaling pathway (**Figure 4**), suggesting that the JAK2-STAT3 pathway is one of the main potential signaling pathways of YQHY induced ferroptosis in GC.

#### 3.1.5 Molecular Docking

**Figure 5:**
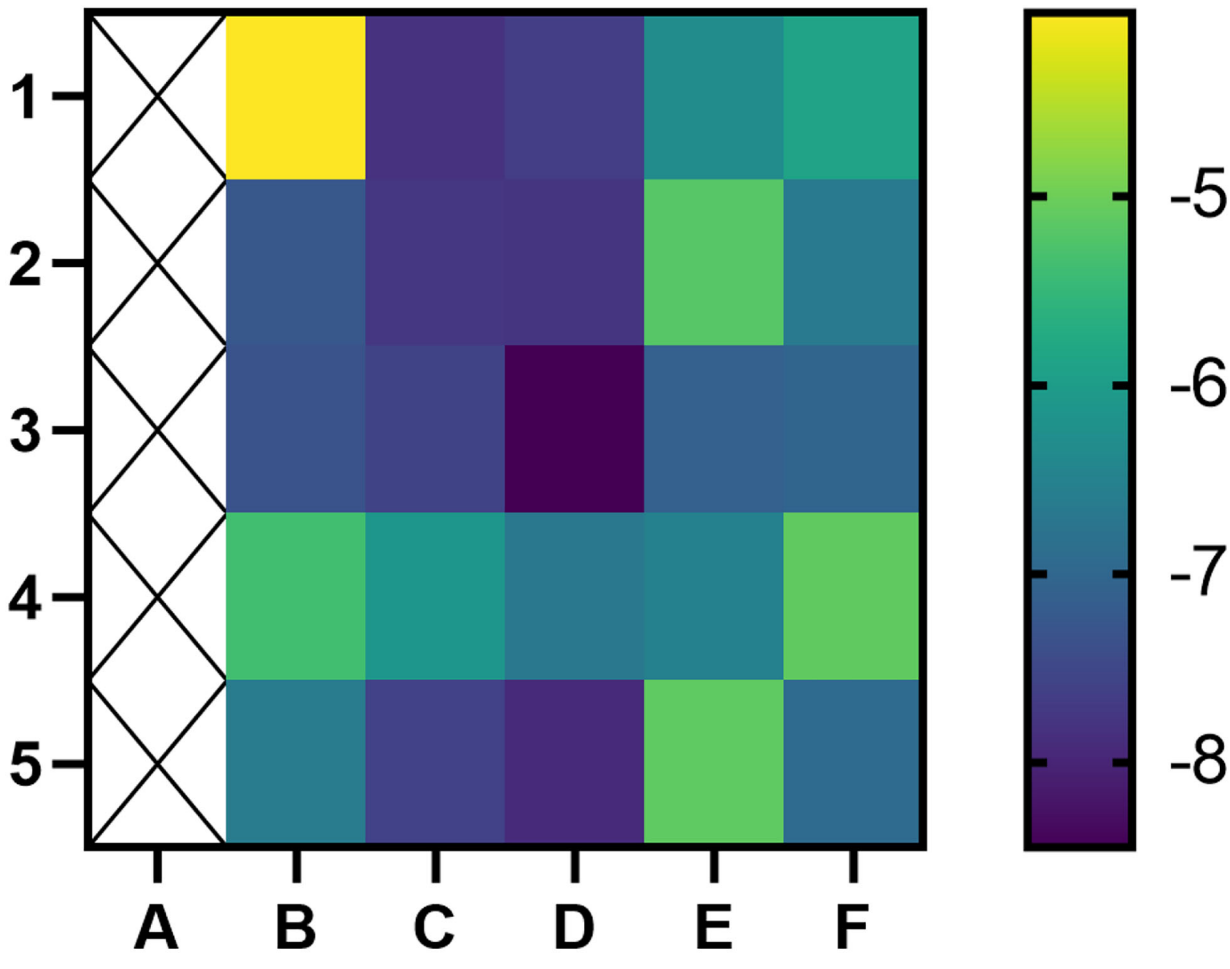
Heatmap of binding between main active components and hub targets. The darker the color, the better the binding activity. **(A)** represent blank, **(B–F)** represent TP53, ATM, SMAD4, PTGS2, and ACSL4, respectively, while 1, 2, 3, 4, and 5 represent Quercetin, DIBP, DBP, Mipax, and Phaseol, respectively.

**Figure 6:**
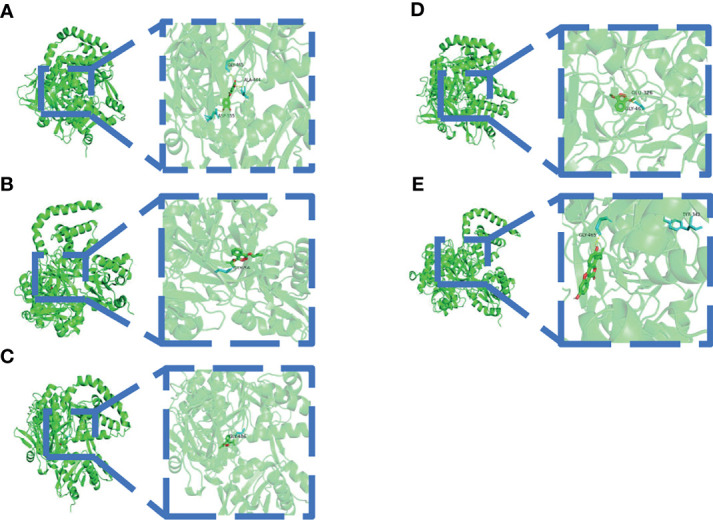
Schematic diagram of docking between active compounds and ACSL4. **(A–E)** represent the molecular binding of ACSL4 with Quercetin, DIBP, DBP, Mipax, and Phaseol, respectively.

A binding energy <−5 kcal mol^−1^ indicates good binding activity. The results of molecular docking were plotted as a heatmap (**Figure 5**). It can be seen from the figure that DIBP, DBP, Phaseol with TP53, SMAD4, and ACSL4 all had good binding activities. We selected the molecular docking results of ACSL4 with the main active compounds, and visual analysis was carried out by PyMol software (**Figure 6**).

### 3.2 Bioinformatics Analysis

**Figure 7:**
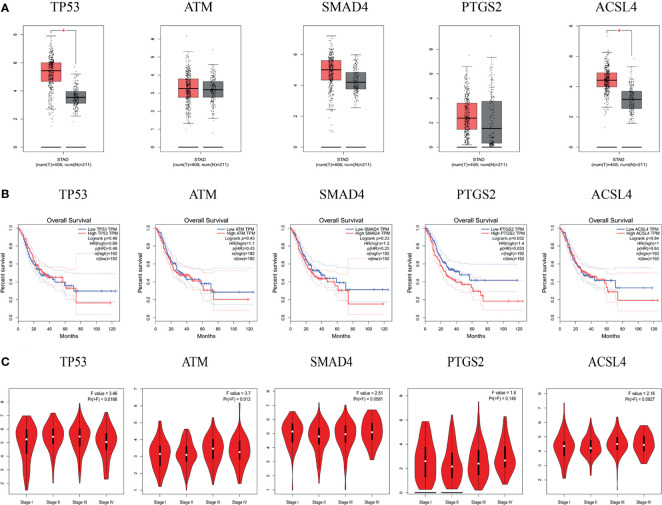
mRNA expression level, pathological stage and OS in the GEPIA database. **(A)** Box plots showing the mRNA expression levels of TP53, ATM, SMAD4, PTGS2, and ACSL4. Red represents Tumor, Gray represents normal. **(B)** The line charts show the OS of hub genes in GEPIA. The survival curve comparing the patients with high (red) and low (blue) expression in GC. **(C)** The violin diagram shows the stage plot of mRNA expression level and pathological stage in the GEPIA database.

The results of the GEPIA database showed that the mRNA levels of TP53 and ACSL4 were significantly highly expressed in GC tissues (**Figure 7A**). Survival analysis of the hub targets (**Figure 7B**) showed that the prognostic value of PTGS2 was significantly different (P <0.05). We analyzed the relationship between hub target mRNA levels and the pathological stage of GC. The results showed that the levels of ATM changed significantly with pathological stage and increased significantly in stage IV (**Figure 7C**).

**Figure 8:**
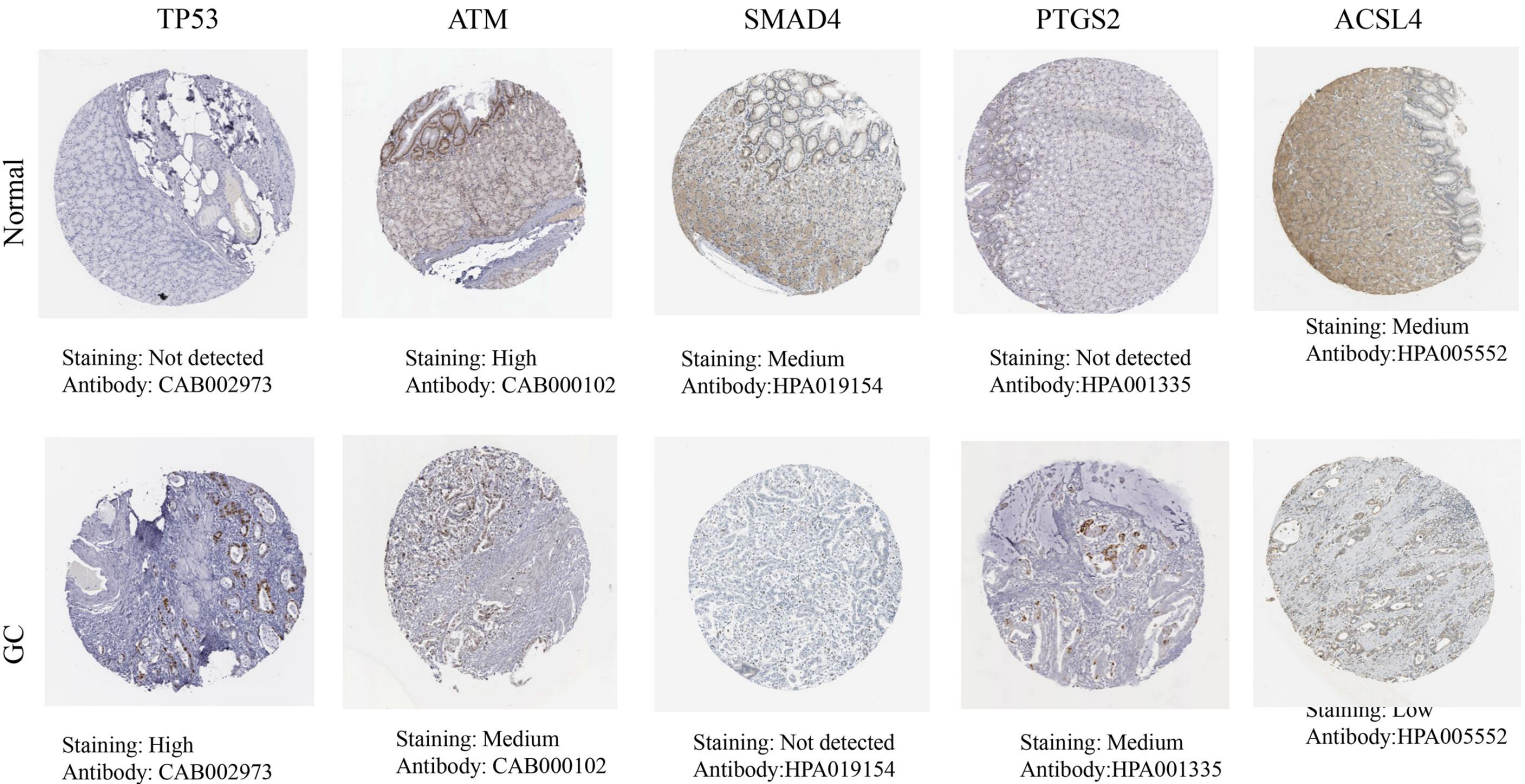
The protein expression levels in the HPA database.

The HPA database results showed that the hub targets were expressed to different degrees in normal gastric tissues. Compared with normal gastric tissues, the expression levels of TP53 and PTGS2 were increased in GC tissues, while the expression of ATM, SMAD4, and ACSL4 was decreased in GC tissues (**Figure 8**).

**Figure 9:**
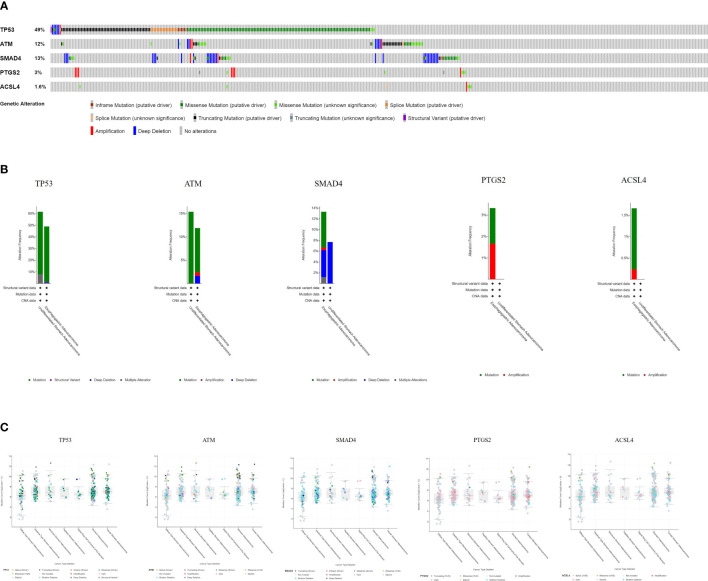
Genetic information of hub targets. **(A)** Data from TCGA of gastric adenocarcinoma showed that 278 of 434 patients (64%) had genetic mutations in these five targets. **(B)** The diagram shows the genetic variation of five targets. **(C)** The diagram shows the number of gene mutations in different types of GC.

The cBioPortal tool showed that 278 of 434 patients (64%) had genetic mutations in these five targets (**Figure 9A**). An overview of the genetic variation of five targets was also analyzed (**Figure 9B**). **Figure 9C** shows the number of gene mutations in different types of GC.

### 3.3 Experimental Validation *In Vitro*

#### 3.3.1 Cell Viability Assay

**Figure 10:**
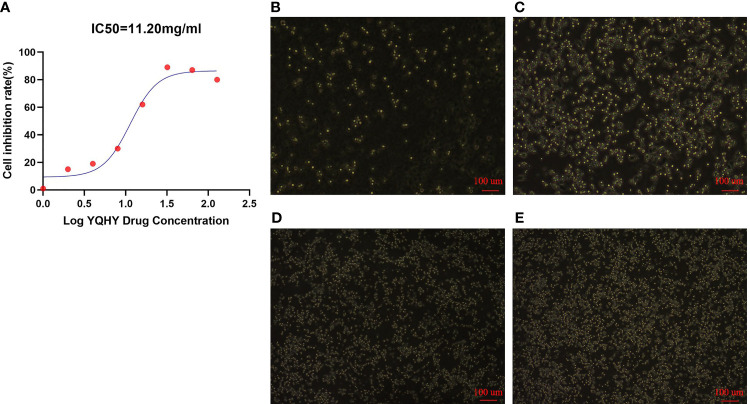
The graph of YQHY inhibiting AGS growth. **(A)** The IC50 of YQHY on AGS cells was 11.20 mg/ml, which means that approximately 50% of AGS cells were inhibited after exposure to 11.20 mg/ml YQHY for 24 h. **(B–E)** show the growth state of AGS cells in different groups after 24 h of dose. Among them, **(B)** represents the YQHY group, **(C)** represents the YQHY + liproxstatin-1 group, **(D)** represents the control group, and **(E)** represents the liproxstatin-1 group.

It can be concluded that with increasing concentrations of YQHY, the toxic effect on AGS gradually intensified, and the inhibition rate of AGS showed a gradually increasing trend (**Figure 10**). According to the curve in the figure, we found that the IC50 of YQHY on AGS was 11.20 mg/ml, which means that approximately 50% of AGS was inhibited after exposure to 11.20 mg/ml YQHY for 24 h. This result suggested that YQHY had a toxic effect on AGS, and we chose this concentration at half inhibition rate for subsequent experiments.

#### 3.3.2 Lipid Peroxidation and GSH Assays

**Figure 11:**
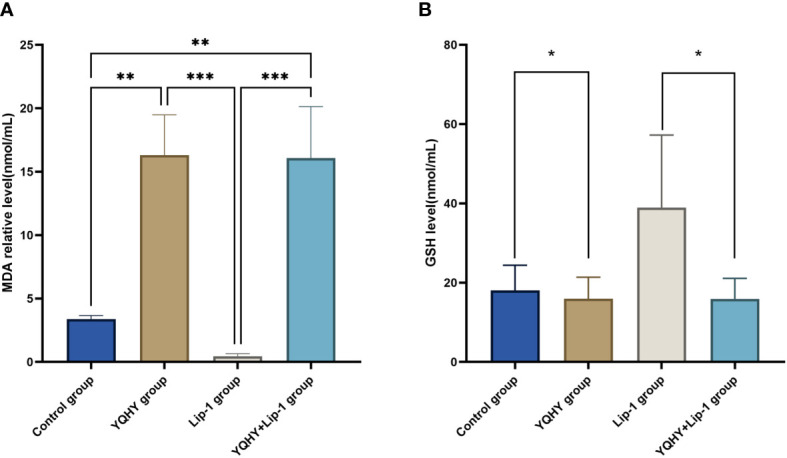
Effect of YQHY on MDA and GSH content in AGS cells (*P <0.05), ** P < 0.01, *** P < 0.001

The MDA content in the YQHY group was the highest compared with the other three groups (P <0.05) (**Figure 11A**), and YQHY reversed the ferroptosis inhibition induced by the ferroptosis inhibitor liproxstatin-1. The GSH content in the YQHY group was the lowest compared with the other three groups (P <0.05) (**Figure 11B**), and the GSH content in the YQHY + liproxstatin-1 group was lower than that in the control group, indicating that YQHY can reduce the intracellular GSH content and induce ferroptosis in AGS and that YQHY can reverse ferroptosis due to liproxstatin-1 inhibition.

#### 3.3.3 Quantitative Real-Time Polymerase Chain Reaction (qRT-PCR)

**Figure 12:**
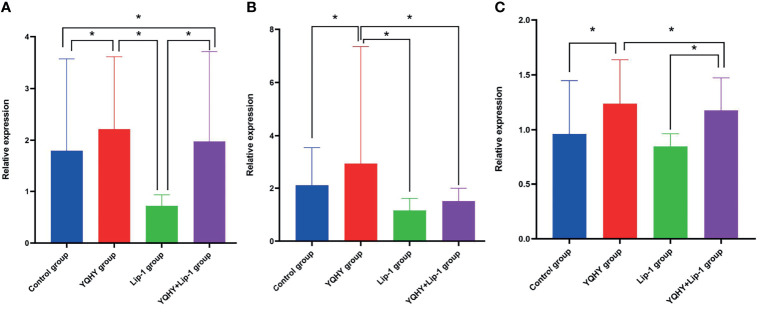
Effect of YQHY on JAK2, STAT3, and ACSL4 mRNA. **(A)** represents the relative mRNA expression of JAK2, **(B)** represents the relative mRNA expression of STAT3, and **(C)** represents the relative mRNA expression of ACSL4. (*P < 0.05).

We used qRT-PCR to measure the effect of YQHY, liproxstatin-1, and YQHY + liproxstatin-1 on the mRNA expression of JAK2, STAT3, and ACSL4. The results showed that the mRNA expression levels of JAK2, STAT3, and ACSL4 were higher in the YQHY group than in the control group (P <0.05). After the addition of liproxstatin-1, the mRNA expression of JAK2, STAT3, and ACSL4 was decreased compared with that in the YQHY group (P <0.05). The mRNA expression of JAK2, STAT3 and ACSL4 in the liproxstatin-1 group was significantly decreased relative to the control group (P <0.05) (**Figure 12**).

#### 3.3.4 Western Blot

**Figure 13:**
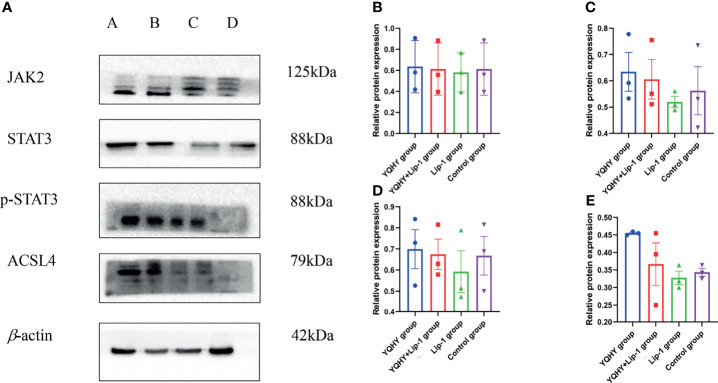
Effect of YQHY on JAK2, STAT3, p-STAT3, and ACSL4 protein. **(A)** A represents the YQHY group, B represents the YQHY + liproxstatin-1 group, C represents the liproxstatin-1 group, and D represents the control group. **(B)** represents the relative protein expression of JAK2, **(C)** represents the relative protein expression of STAT3, **(D)** represents the relative protein expression of p-STAT3, and **(E)** represents the relative protein expression of ACSL4.

Compared with the control group, the protein expression of JAK2, STAT3, p-STAT3, and ACSL4 in the YQHY group was higher than those in the other three groups, while the expression level of each protein in the liproxstatin-1 group was lower. It could be concluded that YQHY can induce ferroptosis in AGS by regulating the JAK2-STAT3 pathway and the expression of ACSL4 (**Figure 13**).

## 4 Discussion

Recently, the incidence and mortality of GC have gradually increased, which is related to diet, lifestyle, genetics, and other factors. Due to distant metastasis and recurrence, most patients have a poor prognosis (22). According to clinical studies, YQHY has a clear antirecurrence and metastasis effect in GC (23). Our team wanted to further investigate whether this is achieved by inducing ferroptosis in GC and whether the influence of the JAK2–STAT3 signaling pathway and ACSL4 expression is one of the mechanisms of YQHY-induced ferroptosis in GC.

Through the network pharmacology study, Quercetin, DIBP, DBP, Mipax, and Phaseol were identified as the main active compounds of YQHY-induced ferroptosis in GC. Quercetin is a flavonoid, which exists in many edible and medicinal plants (24). It has various pharmacological activities, such as antioxidant, anti-inflammatory, and antitumor activities. Quercetin can be used as an antioxidant activity ROS scavenger and metal chelating agent to protect gastric epithelial cells from oxidative damage (25, 26). Many studies have shown that quercetin has antiproliferative and antiangiogenic effects in various cancers (27), such as lung cancer (28), breast cancer (29), colon cancer (30), and GC (31). Quercetin was found to arrest cell division by inducing cell cycle arrest at G0/G1 or G2/M phase in BGC-823 gastric cancer cells through protein analysis of Bax, Bcl-2, and caspases (32). Phasol is rich in flavonol glycosides and coumarin. Coumarin can induce apoptosis of Jurkat cells by inducing mitochondrial depolarization, which may contribute to cell death by inducing cell cycle arrest at the G1 phase, reducing BCL2 levels, and increasing PARP-1 cleavage (33), but there have been no reports on its association with GC. DIBP, DBP, and Mipax were the main compounds we identified for the treatment of GC, but there is no related report.

We analyzed the biological functions and signaling pathways of YQHY and found that TP53, ATM, SMAD4, PTGS2, and ACSL4 were the hub targets of YQHY induced ferroptosis in GC. TP53 is a tumor suppressor gene that has attracted wide attention at present. Mutant TP53 has an influence on the proliferation, migration, survival, and invasion of tumors and the drug resistance of chemotherapy drugs (34). When TP53 is mutated, the original tumor suppressor function is affected, which further increases the apoptosis process of gastric mucosa cells and impairs the damage repair function of the cells, thus inducing the transition of normal cells to cancer cells and increasing the incidence of GC (35). ATM is a cell housekeeping gene encoding ATM protein kinase, which plays a key role in the early signal transduction of cell cycle checkpoints (36, 37). ATM is a potential marker for GC (38), and previous studies (39) have confirmed that HP infection can cause upregulated expression of ATM. SMAD4 is a tumor suppressor gene discovered in recent years. Patients with GC often have mutations or loss of expression of SMAD4 (40). Reduced expression of SMAD4 in GC is one of the main reasons for its abnormal proliferation and migration (41). PTGS2 is closely related to apoptosis and promotes tumor progression (42). Lin (43) demonstrated that PTGS2 mediated cisplatin-induced BCL2 expression and subsequent apoptosis resistance through a PGE2/EP4/MAPK (ERK1/2, P38)-dependent mechanism. The ACSL family mainly catalyzes fatty acids with 12–20 carbon chains (44). Evidence has shown that ACSLs are enzymes crucial for the body’s response to fatty acid metabolism (45). Mammalian ACSLs have five different isozymes, namely, ACSL1, ACSL3, ACSL4, ACSL5, and ACSL6, which are different in intracellular functions in their tissue expression specificity (46). Studies have shown that ACSL4 is involved in various biological processes, such as proliferation, apoptosis, migration, and invasion of tumor cells. It is also a biomarker and contributor of ferroptosis (47). Ferroptosis contributes to the antitumor function of several tumor suppressors, such as p53, BAP1, and fumarase (48). ACSL4 was differentially expressed in tumor tissues compared with adjacent carcinoma normal tissues. For example, an increase in ACSL4 expression is related to the differentiation of colon adenocarcinoma (49). One of these reports indicated that ACSL4 is part of the mechanism responsible for the promotion of breast cancer cell proliferation, invasion, and migration (50). However, ACSL4 is underexpressed in GC tissue and represents an undifferentiated or poorly differentiated malignant phenotype (51). Therefore, the expression of ACSL4 can inhibit the migration and invasion of GC, which illustrates that ACSL4 can be a potential therapeutic target as a tumor suppressor in GC.

The enrichment analysis of the KEGG suggested that the JAK2–STAT3 pathway was one of the main potential signaling pathways of YQHY induced ferroptosis in GC. The JAK2–STAT3 signaling pathway is a typical oncogenic signaling pathway (52). JAK2 serves as a signaling hub that integrates extracellular signals from interleukin receptors and oncogenic receptor tyrosine kinases into STAT3, which phosphorylates STAT3 at Y705 and homodimerizes with p-STAT3 to induce its nuclear translocation and transcriptional activity through interaction with its phosphorylated Y705 site and SH2 domain (53, 54). STAT3 binds to the promoters of its target genes to induce tumor cell migration, growth, and differentiation and plays an important role in the development of a variety of tumors (55).

Molecular docking results showed that the main active compounds of YQHY had good binding activity with the hub targets, and then we identified the hub targets in different databases. We found that the mRNA levels of TP53 and ACSL4 were significantly expressed in GC tissues, and the prognostic value of PTGS2 was significantly different (P b<0.05). The levels of ATM changed significantly with pathological stage and increased significantly in stage IV. The cBioPortal tool showed that 278 of 434 patients (64%) had genetic mutations in these five targets. The results of the above analysis were mostly consistent with previous literature reports.

To further clarify the relationship between YQHY and ferroptosis, we selected ACSL4, which is closely related to ferroptosis, as the target of this topic, and the JAK2–STAT3 pathway as the pathway of this topic to explore whether YQHY could induce ferroptosis in GC by influencing the expression of ACSL4, JAK2, and STAT3. The results showed that YQHY had a toxic effect on AGS, and with increasing concentration, the inhibition rate of AGS showed a gradually increasing trend. The increase in MDA and the decrease in GSH synthesis are the main characteristics of ferroptosis. We found that the MDA content in the YQHY group was the highest compared with the other three groups (P <0.05), and YQHY reversed the ferroptosis inhibition induced by the ferroptosis inhibitor liproxstatin-1. The GSH content in the YQHY group was the lowest compared with the other three groups (P <0.05), and the GSH content in the YQHY + liproxstatin-1 group was lower than that in the control group, indicating that YQHY can reduce the intracellular GSH content and induce ferroptosis in AGS and that YQHY can reverse ferroptosis due to liproxstatin-1 inhibition.

The qRT-PCR results showed that the mRNA expression levels of JAK2, STAT3, and ACSL4 were higher in the YQHY group than in the control group. After the addition of liproxstatin-1, the mRNA expression of JAK2, STAT3, and ACSL4 was decreased compared with that in the YQHY group. The mRNA expression of JAK2, STAT3 and ACSL4 in the liproxstatin-1 group was significantly decreased relative to the control group.Western blot analysis confirmed that compared with the control group, the expression of JAK2, STAT3, p-STAT3, and ACSL4 protein in the YQHY group was higher than those in the other three groups, while the expression level of each protein in the liproxstatin-1 group was lower. It could be concluded that YQHY can induce ferroptosis in AGS by regulating the JAK2-STAT3 pathway and the expression of ACSL4. This illustrates that influencing the JAK2-STAT3 signaling pathway and expression of ACSL4 is one of the mechanisms of inducing ferroptosis in GC, and induction of ferroptosis may be one of the possible mechanisms of YQHY’s anti-recurrence and metastasis of GC.

## 5 Conclusion

The material basis of YQHY induced ferroptosis in GC is based on active compounds such as Quercetin, DIBP, DBP, Mipax, and Phaseol. The related mechanism was characterized by multiple targets and pathways. YQHY can induce ferroptosis in GC by affecting the JAK2–STAT3 pathway and the expression of ACSL4, and induction of ferroptosis may be one of the possible mechanisms of YQHY’s anti-recurrence and metastasis of GC.

## Data Availability Statement

The original contributions presented in the study are included in the article/**Supplementary Material**. Further inquiries can be directed to the corresponding author.

## Author Contributions

PS designed the research. SS analyzed the data and wrote the paper. FW, SG, WH, JL, JZ, PG, SR, XC, and YL selected the materials. All authors read and approved the submitted version.

## Funding

This work was funded by the National Natural Science Foundation of China (nos. 81673918). Pilot GC project of clinical collaboration of traditional Chinese medicine and Western medicine on major difficult diseases in the state administration of traditional Chinese medicine; the 2019” Construction Project of Evidence-based Capacity for Traditional Chinese Medicine” (2019XZZX-ZL003) in state administration of traditional Chinese medicine; the Open Program of the Third Phase of the Program of Traditional Chinese Medicine (TCM) Advantageous Subjects (ZYX03KF020); and the Science and Technology Project of Jiangsu Provincial Administration of Traditional Chinese Medicine (ZD201803).

## Conflict of Interest

The authors declare that the research was conducted in the absence of any commercial or financial relationships that could be construed as a potential conflict of interest.

## Publisher’s Note

All claims expressed in this article are solely those of the authors and do not necessarily represent those of their affiliated organizations, or those of the publisher, the editors and the reviewers. Any product that may be evaluated in this article, or claim that may be made by its manufacturer, is not guaranteed or endorsed by the publisher.

## Abbreviations

GC, gastric cancer; YQHY, Yiqi Huayu Decoction; TCMSP, Traditional Chinese Medicine Systems Pharmacology Database and Analysis Platform; OB, Oral bioavailability; DL, Drug-likeness; GO, Gene Ontology; BP, Biological process; CC, Cellular component; MF, Molecular function; FDR, Error Detection Rate; KEGG, Kyoto Encyclopedia of Genes and Genomes; PPI, Protein–protein interaction; OS, Overall Survival; qRT-PCR, Quantitative Real-Time Polymerase Chain Reaction.
